# A comprehensive review on *Gossypium hirsutum* resistance against cotton leaf curl virus

**DOI:** 10.3389/fgene.2024.1306469

**Published:** 2024-02-19

**Authors:** Sahar Nadeem, Syed Riaz Ahmed, Tahira Luqman, Daniel K. Y. Tan, Zahra Maryum, Khalid Pervaiz Akhtar, Sana Muhy Ud Din Khan, Muhammad Sayyam Tariq, Nazar Muhammad, Muhammad Kashif Riaz Khan, Yongming Liu

**Affiliations:** ^1^ Nuclear Institute for Agriculture and Biology College, Pakistan Institute of Engineering and Applied Sciences (NIAB-C, PIEAS), Faisalabad, Pakistan; ^2^ Pakistan Agriculture Research Council (PARC), Horticulture Research Institute Khuzdar Baghbana, Khuzdar, Pakistan; ^3^School of Life and Environmental Sciences, Plant Breeding Institute, Sydney Institute of Agriculture, Faculty of Science, The University of Sydney, Sydney, NSW, Australia; ^4^ Agriculture and Cooperative Department, Quetta, Pakistan; ^5^ Plant Breeding and Genetics Division, Cotton Group, Nuclear Institute for Agriculture and Biology, Faisalabad, Pakistan; ^6^ National Nanfan Research Institute (Sanya), Chinese Academy of Agricultural Sciences, Sanya, China

**Keywords:** Cotton (*G. hirsutum* L.), CLCuD, genome editing, next-generation technologies, speed breeding

## Abstract

Cotton (*Gossypium hirsutum* L.) is a significant fiber crop. Being a major contributor to the textile industry requires continuous care and attention. Cotton is subjected to various biotic and abiotic constraints. Among these, biotic factors including cotton leaf curl virus (CLCuV) are dominant. CLCuV is a notorious disease of cotton and is acquired, carried, and transmitted by the whitefly (Bemisia tabaci). A cotton plant affected with CLCuV may show a wide range of symptoms such as yellowing of leaves, thickening of veins, upward or downward curling, formation of enations, and stunted growth. Though there are many efforts to protect the crop from CLCuV, long-term results are not yet obtained as CLCuV strains are capable of mutating and overcoming plant resistance. However, systemic-induced resistance using a gene-based approach remained effective until new virulent strains of CLCuV (like Cotton Leaf Curl Burewala Virus and others) came into existence. Disease control by biological means and the development of CLCuV-resistant cotton varieties are in progress. In this review, we first discussed in detail the evolution of cotton and CLCuV strains, the transmission mechanism of CLCuV, the genetic architecture of CLCuV vectors, and the use of pathogen and nonpathogen-based approaches to control CLCuD. Next, we delineate the uses of cutting-edge technologies like genome editing (with a special focus on CRISPR-Cas), next-generation technologies, and their application in cotton genomics and speed breeding to develop CLCuD resistant cotton germplasm in a short time. Finally, we delve into the current obstacles related to cotton genome editing and explore forthcoming pathways for enhancing precision in genome editing through the utilization of advanced genome editing technologies. These endeavors aim to enhance cotton’s resilience against CLCuD.

## Graphical Abstract

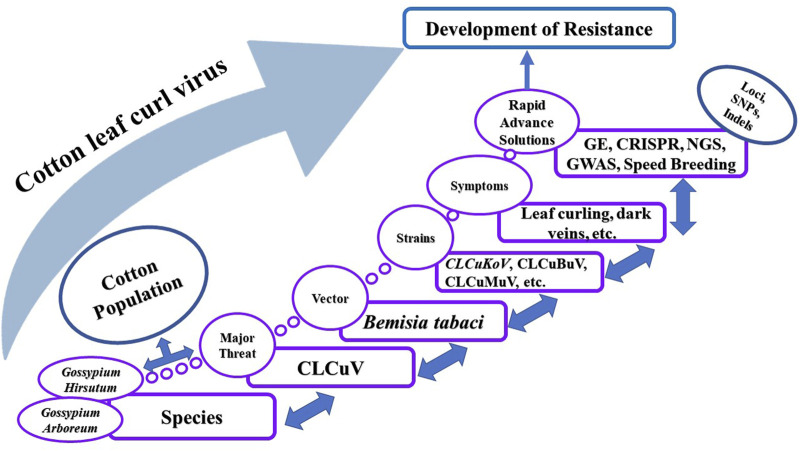

## 1 Introduction

**FIGURE 1:**
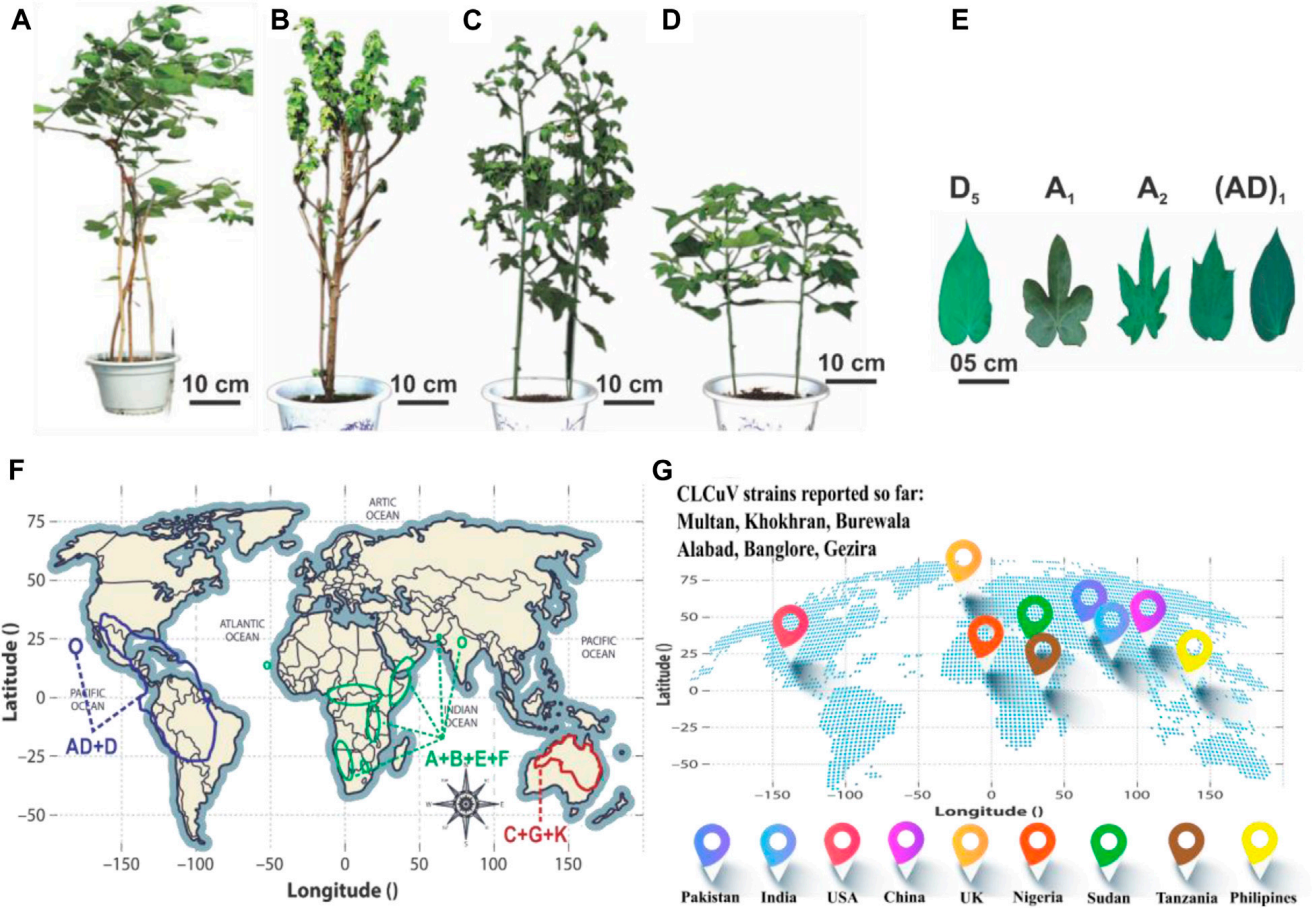
A global perspective on cotton phenotypes, geographic distributions, and the worldwide prevalence of CLCuD. **(A–D)** show the morphology of *G. raimondii*, *G. herbaceum*, *G. arboretum* and *G. hirsutum*, respectively. **(E)** Represents the Cotton leaf shape of different clades. **(F)** The distribution patterns of A to G, K, and AD genomes are depicted geographically. The AD and D genome cluster is represented in yellow, the A, B, E, and F genome clusters in green, and the C, G, and K genome clusters in red. **(G)** Prevalence of different CLCuD strains worldwide.

Cotton (*Gossypium hirsutum* L.) stands as a high-quality fiber-producing plant that contributes greatly to the world’s textile industry by generating an annual economic income of 600 billion US dollars (Ashraf et al., 2018). It serves as a model system for investigating plant polyploidization, cell wall biogenesis, and cell elongation (Lu et al., 2018). The genus *Gossypium* encompasses seven tetraploid species (2n = 4x = 52) and 45 diploid species (2n = 2x = 26), showcasing remarkable morphological diversity (Lu et al., 2018). This variation includes a spectrum of plant structures, spanning from untamed perennial trees and shrubs to cultivated annual herbaceous plants, accompanied by unique fiber traits and diverse leaf shapes. Cotton has captivated the interest of agricultural researchers, evolutionary biologists, and taxonomists for its remarkable genomic diversity and widespread dispersion. This diversity has given rise to the evolution of eight distinct diploid cotton groups, designated as A-, B-, C-, D-, E-, F-, G-, and K-genomes, along with an AD-genome clade. The classification of the genus *Gossypium* into three primary lineages delineated mainly by geographical and morphological factors, including the Australian clade (C-, G-, and K-genomes), the African-Asian clade (A-, B-, E-, and F-genomes), and the New World clade (D- and AD-genomes) (Figure 1) (Ahmed, 1999; Huang et al., 2021). Among these, *G. hirsutum* [(AD)_1_], commonly called upland cotton, currently dominates the global cotton industry by contributing approximately 95% of the natural lint fiber essential for textiles.

The textile industries rely on good quality cotton lint fiber to fulfill market demands. Each clade has a separate fiber lint quality and yield. Therefore, the fiber lint is of great importance in cotton domestication and quality among cotton breeders. The progression of fiber development can be categorized into four successive phases: initiation, elongation, secondary cell wall (SCW) biosynthesis, and maturation, categorized based on the days post-anthesis (DPA) (Prasad et al., 2022). Cotton fibers can be additionally classified into two forms: adherent fuzz fibers, which commence development around 5 to 10 DPA and reach a final length of about 5 mm, and spinnable lint fibers, originating before flowering and growing to a final length of roughly 3 cm.

Pakistan is the fourth-largest producer of cotton following India, China, and the United States of America (Shuli et al., 2018). Unfortunately, cotton production is continuously being challenged by various biotic and abiotic stresses, among which CLCuD is most prevalent. CLCuV has emerged as a prominent threat to cotton, causing major losses of up to 80%–87% in Pakistan and North India (Mansoor et al., 2003; Varma and Malathi, 2003; Sattar et al., 2013; Monga and Sain, 2021; Chauhan et al., 2023). The importance of cotton in the textile industry is undeniable, therefore, requires continuous attention to its improvement. The virus is a major cause of low productivity in cotton and hence, efforts are being made to induce resistance in cotton crop against the disease (Ali et al., 2015). The story of the emergence of CLCuV as a threat is not new. For the first time, it was reported in Nigeria in 1912. Later on, it was documented in Sudan in 1924, followed by occurrences in Tanzania in 1926, and later in the Philippines in 1959. In Pakistan, CLCuD was discovered in 1967 on scattered cotton plants of the *G. hirsutum* species in the Multan district (Hussain and Ali, 1975). However, the disease was not considered a serious threat until the 1990s, when it appeared as an epidemic and disastrously brought a monetary loss of $5 US billion, throughout the world (Briddon and Markham, 1994; Leke et al., 2015)

CLCuV is acquired, carried, and transmitted via whitefly (*Bemisia tabaci*) (Nogia et al., 2014). Once the vector acquires the virus, it is retained by the carrier whitefly throughout its life. Plants infected with CLCuD show a broad range of symptoms ranging from stunted growth, yellowing of leaves, and appearance of dark green thickened veins on leaves followed by upward or downward curling (Heigwer et al., 2016). In case of high disease severity, curled leaves may even develop small outgrowths (also called enations) on their lower surface. Moreover, affected leaves become brittle and leaf size is substantially reduced (Mansoor et al., 1993; Harrison et al., 1997; Qazi et al., 2007). At the seedling stage, CLCuV affects the cotton yield byreducing the cotton boll’s number and size, flowering time, and posing undesirable effects on seed and fiber quality, and final yield (Ali et al., 2013; Ji et al., 2015; Heigwer et al., 2016). It is challenging to fight CLCuV because of the prevalence of active viral strains and greater recombination rates of CLCuV complex. A case study by Qadir et al. (2019), revealed that the recombination potential of CLCuV is significantly higher. A greater recombination rate was observed in all genes (*IR, V1, V2, C1, C4, and C5*) associated with cotton leaf curl Kokhran Virus (CLCuKoV). Furthermore, CLCuKoV also donated in the *C2, C3* regions of cotton leaf curl Multan virus (CLCuMuV). Entirely, these observations clearly show the uniqueness of Indian CLCuMuV isolates representing the contribution of CLCuKoV in all the genes (Qadir et al., 2019). Higher recombination rate of CLCuV leads to multiple strain infection which makes viral control difficult. The presence of various alternative hosts and favorable environmental conditions has further complicated the disease control. Many efforts were made during the 1990s to produce CLCuV resistant varieties, yet the outbreak of another CLCuV strain, namely, Cotton Leaf Curl Burewala (CLCuBuV) broke the resistance and the varieties fell prey to it (Sarwar et al., 2022).

Note: Cotton in Asia, experienced two significant epidemics within this time frame: the ‘Multan epidemic,’ spanning from 1988 to 1999, followed by a period of relative calm. However, in 2002, the ‘Burewala epidemic’ emerged in the cotton fields of the Indo-Pak subcontinent and persisted until 112 2013–2014.

The availability of various alternative hosts like hibiscus, okra, etc., and different farming practices could provide a reservoir of novel sources of new virulent CLCuV strains (Ali et al., 2022). Systemic-induced resistance using a gene-based approach remained effective until a new virulent strain of CLCuV came into existence named Cotton Leaf Curl Burewala Virus (CLCuBV). The damaging effects of the virus could only be overcome by proper management strategies. Many efforts have been used to protect the cotton crop from CLCuD. Disease control by biological means and development of CLCuV resistant cotton varieties are in progress. Use of pathogen and non-pathogenbased approaches and advanced molecular practices can be an effective way to fight viral causative agents (Hasan et al., 2019). We present an insightful overview encompassing the comprehension of the cotton genome, unraveling the genetic architecture of CLCuV, understanding cotton’s defense mechanisms against CLCuD, and exploring the applications of genome editing techniques for disease management, with a focus on cotton. Furthermore, we delineate the broader applications of other innovative technologies such as Next-Generation technologies, functional genomics, and speed breeding in cotton biological research and enhancement. Lastly, we discuss the ongoing challenges related to cotton genome editing and deliberate on the future opportunities involving various strategies and advancements in genome editing tools to foster the sustainability of cotton production.

## 2 Navigating CLCuV vectors: unveiling transmission mechanisms

Over the past three decades, insects from the genus *Bemisia* have become the major devastating pests of agriculture and horticulture (Ribeiro et al., 2003). The most significant and widespread among these are the whiteflies that are associated with *Geminiviruses* or more specifically to *Begomoviruses*. Their transmission may be directly associated with the silver leaf type of the whitefly. *Bemisia tabaci* is an indiscriminate feeder, facilitating rapid and proficient spread of CLCuV from affected plant hosts to neighbouring crops (Brown and Czosnek, 2002). Among all damaging pests of cotton, whitefly is responsible for about 50% yield loss and is a major restraint of boll formation (Mansoor et al., 2003). Intensive escalation in the whitefly population is because of the immense propagation of diseases by viruses through whiteflies.

*Begomoviruses* exist in either mono-partite or bi-partite forms. Bi-partite *Begomoviruses* contain both DNA A and DNA B, associated with New World pathogens. On the other hand, mono-partite *Begomoviruses* only contain a circular single-strand DNA-A and two satellite molecules viz., α and β satellite, classified as an essential component for disease severity in Old World pathogens (Hasan et al., 2019). An example of a mono-partite particle is CLCuV (Liu et al., 1998; Mansoor et al., 1999). PCR amplification of CLCuD-infected cotton indicated the presence of CLCuV (Fiallo-Olivé and Navas-Castillo, 2023). CLCuV possesses a circular single-stranded deoxyribonucleic acid (ssDNA) molecule, tightly encapsulated inside a geminate particle. Previous research has shown a wide range of hosts for mono-partite and bi-partite *Begomoviruses* (Yazdani-Khameneh et al., 2016). As the most destructive pathogens, CLCuVs are widely spread in Central and South-East Asia. The viruses carry distinct recombinations to break down transgenic resistance in cotton cultivars (Devendran et al., 2022).

### 2.1 Geminiviruses

*Geminivirus* is a group of pathogenic viruses transmitted by insects. The family *Geminiviridae* is responsible for causing several diseases in plants around the globe (Arif et al., 2022). *Geminiviruses* contribute to the largest group of plant viruses. Zerbini and colleagues (2017), categorized *Geminiviruses* into nine genera, utilizing criteria such as genome arrangement, transmission vectors, and host plants. This classification comprises *Turncurtovirus*, *Topocuvirus*, *Mastrevirus*, *Grablovirus*, *Eragovirus*, *Curtovirus*, *Capulavirus*, *Begomovirus,* and *Becurtovirus* along with a subset of species that remain unclassified (Zerbini et al., 2017). Recently, this family has been increased from nine recognized genera to fourteen genera (Materatski et al., 2021). *Begomoviruses* is the largest group of *Geminiviruses* with around 520 accepted viral species ((Uniyal et al., 2019; Ouattara et al., 2022; Fiallo-Olivé and Navas-Castillo, 2023).

CLCuV *Geminiviruses* are responsible for the transmission of CLCuV through whiteflies. These viruses are taken up by whiteflies when they feed on infected plants and are then transmitted to healthy plants during subsequent feeding. This transmission mechanism makes *Geminiviruses* key contributors to the spread and persistence of CLCuV in cotton crops, leading to significant agricultural and economic impacts (Jain et al., 2023). Significant crop losses (such as high plains viral disease of wheat and corn cause by wheat mosaic virus and maize red stipe virus, sorghum mosaic virus and sugarcane mosaic virus cause by potyvirus, and rice stripe virus cause and transmitted by *Laodelphax striatellus*) have been reported due to various disease infestations leading to viral diseases spread worldwide (Lu et al., 2021; Tatineni and Hein, 2021; Xu, et al., 2021). Major reasons that resulted in disease epidemics include the recombination of many different *Geminiviruses* (i.e., the exchange of genetic materials among at least two *Geminiviruses* to become more virulent) that co-infect the same crop, expansion, and development of agriculture in newer areas, or transport of infectious plant materials to other regions. Additionally, plant co-infections can be spread by vectors that pick up the pathogens from plants infected with multiple diseases, either from different plants one after another, or by different vectors each carrying a different disease. Moreover, the migration of carrier insect vectors from one location to another also brings epidemic explosion (Gray and Banerjee, 1999).

### 2.2 Begomoviruses

The genus *Begomovirus* is a serious threat not only to cotton but also to other crops such as tomato and cassava (Donnelly and Gilligan, 2023; Fortes et al., 2023). *Begomovirus* behaves the same way as *Geminiviruses* in transmitting the virus. The complex interaction between *Begomoviruses*, whiteflies, and cotton plants highlights the significance of understanding and managing these factors to mitigate the impact of CLCUV on cotton crops. Bi-partite begomoviruses are comprised of DNA-A and DNA-B, each approximately 2,600 nucleotides long (Fondong, 2013). The mono-partite particles contain single DNA-A molecule of about 2,800 nucleotides (Fauquet and Stanley, 2003). *Betasatellites* encompass circular, single-stranded DNA molecules comprising around 1,350 nucleotides, responsible for disease inducing symptoms (Zhou et al., 2003; Saunders et al., 2004). *Alphasatellites* are also circular single-stranded DNA made up of approximately 1,375 nucleotides and associated with a rolling-circle replication (RCR) initiator protein.

## 3 Decoding CLCuV genome: unveiling the genetic architecture

### 3.1 DNA A

**FIGURE 2:**
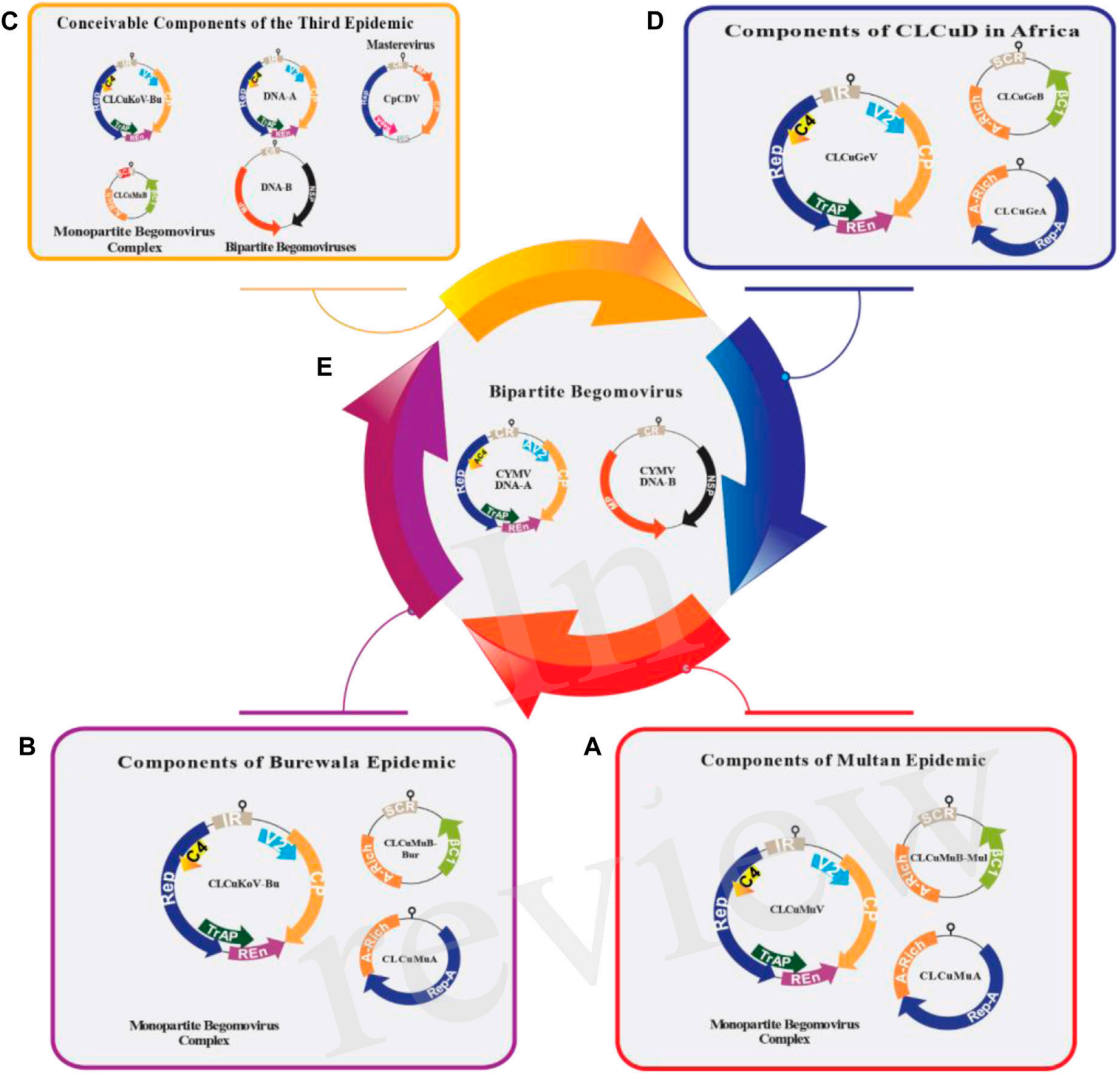
The genomic arrangement of all the contributing viruses responsible for CLCuD in Asia **(A–C)** and Africa **(D,E)**. The prominent *Begomovirus*, along with DNA satellites associated with the suggested third epidemic **(C)**, Burewala epidemic **(B)** and Multan epidemic **(A)** are displayed. In the period of the Multan epidemic, various *Begomoviruses* were simultaneously identified in cotton plants, including tomato leaf curl Bangalore virus (ToLCBaV), Papaya Leaf Curl Virus (PaLCuV), cotton leaf curl Kokhran virus (CLCuKoV) and cotton leaf curl Alabad virus (CLCuAlV). Nevertheless, the primary.

CLCuV is an example of a mono-partite particle that contains single-stranded DNA (Briddon et al., 2000). DNA-A of mono-partite particles encodes for viral DNA replication, insect transmission and control gene expression (Noueiry et al., 1994). DNA-A has six open reading frames (ORFs) that encode various proteins (Yaqoob et al., 2020). In the virion sense, there are two ORFs, namely, AV2 (encodes the movement of a protein; also called precoat) and AV1 (encodes the coat protein), while in the complementary sense, four ORFs [AC4 (determines the expression of symptoms), AC3 (encodes replication enhancer protein), AC2 (encodes a transcription activator protein), and AC1 (encodes the inititation protein called Rep)] are found (Marwal et al., 2014). The genomes of mono-partite viruses and the DNA-A component of bi-partite *Begomoviruses* contain the instructions for the coat protein (CP) and V2 (also known as AV2) in the orientation corresponding to the virion sense (Figure 2). The coat protein is involved in virus proliferation, vector transmission, disease virulence and encapsidation (Singh et al., 2021). AV2 is associated with cell-to-cell movement and pathogenicity (Arif et al., 2022). The proteins linked to replication (AC1/C1 or Rep protein), transcriptional activation (AC2/C2 or TrAP protein), replication enhancement (AC3/C3 or REn protein), and the AC4/C4 protein are encoded in the orientation corresponding to the complementary sense (Fondong, 2013; Zhou, 2013). AC1 serves viral replication and gene expression whereas AC2 is responsible for transcription activators of rightward ORFs and suppressors of post-transcriptional gene silencing (PTGS) (Dilip, 2016; Sharma and Prasad, 2020). AC3 is involved in viral replication and symptom development and AC4 in gene silencing (Faiz and Abhinav, 2021; Roumagnac et al., 2021). DNA-B consists of two ORFs, BC1 in the complementary sense and BV1 in the virion sense. BV1 plays a role in nuclear trafficking and BC1 encodes movement protein MP, involved in inter- and intracellular movement (Diamos et al., 2019; Yadav et al., 2021).

### 3.2 DNA B

The genome component DNA-B (absent in CLCuV but present in bi-partite *Begomoviruses*) encode the movement protein (MP) in the complementary-sense orientation and nuclear shuttle protein (NSP) in the virion sense (Sahu et al., 2014). DNA-B has two open reading frames and encodes proteins associated with inter and intracellular movements (Mubarik et al., 2021). An intergenic intronic region exists between ORFs of the virion and complementary-sense regions that contain a hair-pin loop-like structure and cis-acting regulatory elements essential for gene expression. The loop-like structurecontains a nona-nucleotide conserved sequence ‘TAATATTAC’ and small repetitive sequences ‘iterons’ that provide sequence-specific binding sites for Rep (C_1_/AC_1_) protein. Both iterons and hairpin loop form the origin of replication (*ori*) for initiation of viral DNA replication (Zhou, 2013).

### 3.3 DNA α-satellite and β-satellite

*Alpha* and *betasatellites* are assumed to function in encapsulation, insect mediated transmission and cellular movement inside the infective host plants Gnanasekaran and Chakraborty, 2018). Additionally, betasatellites impact symptom development and boost the pathogenicity of helper viruses by elevating viral DNA levels in host plants and inhibiting the plant’s antiviral defense mechanisms (Gnanasekaran et al., 2019). Satellite molecules share a conserved loop like structure with DNA-A. Genome size of these molecules are about 1.4 kb (Azeem et al., 2022). Both satellites contain conserved structures which share broad characteristics. Furthermore, the molecules comprise an “*Ori*” region separated by a non-nucleotide sequence “NANTATTAC” at the apex of the hairpin structure (Rosario et al., 2012). Though, *betasatellites* contain a nonanucleotide motif “TAATATTAC”, observed in a large number of *Begomoviruses* whereas *alpha satellites* contain “TAGTATTAC” motif, seen in various members of *Nanoviridae* family. *Alphasatellite* is capable of replicating itself autonomously. Both *alpha* and *betasatellites* are rich in adenine residues which function in proficient encapsulation and aid in systemic movement of the virus. Both molecules encode a single protein. The α-satellite encodes a Replication protein (RP) associated with rolling circle replication inside the host cells. On the contrary, helper *Begomovirus* is required for *betasatellite* replication. The *β-satellite* also encodes *βC1* protein which is multi-functional and significant for inducing pathogenicity (Zhou, 2013).

*Begomovirus* responsible was for Cotton Leaf Curl Multan Virus (CLCuMuV). In the case of the Burewala epidemic, a recombinant cotton leaf curl Multan betasatellite-Burewala strain (CLCuMuB^Bur^) and a truncated transcriptional activator protein (TrAP) were observed, incorporating approximately 98 nucleotides from the SCR region acquired from Tomato leaf curl betasatellite (ToLCB) (B). The third epidemic in Asia presents several possible scenarios: i) it could involve Cotton leaf curl Kokhran virus-Burewala (CLCuKoV-Bu), which retains an intact TrAP, along with Cotton leaf curl Multan betasatellite (CLCuMuB), containing a ∼24 nt stretch from tomato leaf curl betasatellite (ToLCB), ii) Alternatively, it might include tomato leaf curl New Delhi virus (ToLCNDV) (comprising both DNA-A and DNA-B) and tomato leaf curl virus (ToLCV) (with DNA-A), possibly in conjunction with ToLCNDV (DNA-B), iii) Another potential contributor could be Mastrevirus chickpea chlorotic dwarf virus (CpCDV) (C). Concurrently, the CLCuD situation in Africa comprises: (i) a mono-partite *Begomovirus* complex (D), a recently identified bipartite *Begomovirus*, cymbidium mosaic virus (CYMV), with DNA-A and DNA-B (E). The DNA-A of mono-partite *Begomoviruses* encodes two open reading frames (ORFs) in the virion-sense orientation, specifically the V2 protein and the coat protein. In the complementary-sense orientation, there are four ORFs: the replication-associated protein (Rep), TrAP, replication enhancer protein (REn), and C4 protein (with functions yet to be fully understood). Bi-partite *Begomovirus* DNA-A resembles the mono-partite genome but includes a second genomic component, DNA-B, which encodes two ORFs: the movement protein (MP) and the nuclear shuttle protein (NSP), in both complementary-sense orientations and virion sense. The alphasatellite encodes a single ORF (Rep-A) for autonomous replication, while the betasatellite encodes its single ORF, βC1, in the complementary-sense orientation. βC1 plays a role in assisting the helper *Begomovirus* in various functions.

## 4 Defense mechanisms of cotton against CLCuV

Besides viruses, several other organisms including insects such as cotton aphids, *Hemipteran* sucking insects, cotton bollworms, and Lepidopteran chewing caterpillars are commonly observed in cotton fields. Cotton bollworm predation triggers the activation of genes related to the gibberellic acid (GA) (*GhMPK11*, *SLR1*), ethylene (*GhWRKY70D13*, *GhERF91*), and jasmonic acid (JA) (*GhWRKY70D13*, *JAZ3*) pathways, while simultaneously suppressing genes associated with the salicylic acid pathway within cotton plants (Kumar et al., 2016; Wang et al., 2016; Xia et al., 2018; Xiong et al., 2020; Kundu et al., 2023; Nurimanguli et al., 2023). JA acts as a regulator in facilitating insect defense, working in conjunction with its corresponding target (SPL9) and miR156 (Arora and Chaudhary, 2021). The response to JA is swift in young plants but progressively slows as plants age, displaying an age-related pattern inversely linked to SPL9 group protein levels. Moreover, not all but some of the herbivorous insects have also developed intricate strategies, such as the discharge of effector molecules into the host and the synthesis of diverse detoxification enzymes, to overcome the resistance mechanisms of their host plants. The defense mechanisms of cotton against CLCuD are multifaceted and include both pathogen-derived and non-pathogen-derived strategies. The most significant defense mechanism is the presence of resistance (R) genes in plants (Table 1). These genes provide specific resistance against particular viruses by inducing cell death around infected plant cells, thereby preventing the movement of the virus. Cotton has two associated R genes, *R1CLCuDhir* and *R2CLCuDhi*, which confer resistance against CLCuD (Khan et al., 2015). Furthermore, the exclusive protein βC1, encoded by the satellite, has been demonstrated to hinder the ubiquitination process of the host plant (Gnanasekaran et al., 2019). It achieves this by interacting with the SKP1/CUL1/F- box complex, disrupting plant hormone signaling (Zhang Y. et al., 2019). This disruption ultimately leads to the buildup of the virus and the clustering of symptoms in the host plants. Moreover, βC1 also disrupts the plant’s autophagy pathway by obstructing the normal function of autophagy-related protein 3 and cytosolic glyceraldehyde-3-phosphate dehydrogenase within the host cells (Ismayil et al., 2020).

**TABLE 1:** List of genes associated with CLCUD and other biotic and abiotic factors, directly and indirectly, involved in cotton infections.

Sr. No	Gene ID/Accession no.	Gene symbol	Gene description	Chromosomal position/locus tag	Gene function	References
1	30144432	AV1	Coat protein	BMU10_gp3	Virus proliferation, vector transmission, disease virulence and encapsidation	Singh et al. (2021)
2	30144433	AV2	Pre-coat protein	BMU10_gp2	Involved in Pathogenicity and cell to cell movement	Sodha et al. (2022)
3	30144436	AC1	Replication associated protein	BMU10_gp6	Viral replication and gene expression	Sharma and Prasad. (2020)
4	30144435	AC2	Transcription activator protein	BMU10_gp5	Responsible for transcription activators of rightward ORFs. Suppressor of post-transcriptional gene silencing (PTGS)	Dilip (2016)
5	30144434	AC3	Replication enhancer protein	BMU10_gp4	Viral replication and symptom development	Roumagnac et al. (2021)
6	30144437	AC4	Regulatory protein	BMU10_gp7	Gene silencing	Faiz and Abhinav. (2021)
7	80557347	βC1	BetaC1 protein	QKT41_gp1	Symptom determinant, interact with other helper viruses, affect number of plant species including cotton	Iqbal et al. (2012)
8	988208	ac5	AC5 protein	ToLCNDVsAgp4	Viral replication	Amudha et al. (2010)
9	107958459	WRKY4 0	Probable WRKY transcription factor 40	Chromosome A05/ERO13_A05G119 500v2	Regulate cotton defense for controlling whitefly infestation	Li et al. (2016)
10	107924381	MPK	Mitogen-activated protein kinase kinase 3	Chromosome A11	Silencing of MPK leads to enhanced whitefly susceptibility	Li et al. (2016)
11	988213	BV1	Nuclear shuttle protein	ToLCNDVsBgp1	Role in nuclear trafficking	Diamos et al. (2019)
12	2767401	BC1	BC1 protein	TYLCKV_sBgp2	Encodes movement protein MP, involved in inter- and intracellular movement	Yadav et al. (2021)
13	—	*R1CLCuDhir*	Resistance gene	—	Provides resistance to cotton against CLCuD	Hagan et al. (2019)
14	—	*R2CLCuDhir*	Resistance gene	—	Provides resistance to cotton against CLCuD	Hagan et al. (2019)
15	—	*SCLCuDhir*	suppressor gene	—	Suppressor of resistance to CLCuD	Hagan et al. (2019)
16	107934102	LOC107 934102	Probable zinc metallopeptida se EGY3, chloroplastic	ERO13_A01G009 420v2/Chromosome A01	Chloroplast development. Copes with stress	Naqvi et al. (2017)
17	107928973	LOC107 928973	Probable zinc metallopeptida se EGY3, chloroplastic	ERO13_D01G009 000v2/Chromosome D01	Development and stress response	(Naqvi et al. 2017)
18	105786625	LOC105 786625	Probable zinc metallopeptida se EGY3, chloroplastic	B456_002G01100 0/chromosome 1	Probable membrane-associated metalloprotease that may be involved in chloroplast development.	Marino and Funk. (2012)
19	107909622	LOC107 909622	Chaperone protein dnaJ 16	ERO13_D02G166 200v2/chromosome D02	Pathogen defense, vital in antiviral defense	Verchot (2012), Park and Seo (2015)
20	105802570	LOC105 802570	Transcription factor UNE10	B456_007G08830 0/chromosome 11	Antiviral defense	Wang et al. (2015)
21	107911889	LOC107 911889	Transcription factor UNE10- like	ERO13_A11G078 200v2/chromosome A11	Antiviral defense	Wang et al. (2015)
22	107904753	LOC107 904753	Protein REVEILLE 7	ERO13_D12G273 200v2/chromosome D12	Plant growth contributes to response against pathogens	Wang et al. (2015), Zhong et al. (2016)
23	107902644	LOC107 902644	Transcription factor RADIALIS	ERO13_D05G196 766v2/Chromosome D05	Plant defense response	Katiyar et al. (2012)
24	107951700	LOC107 951700	Putative disease resistance protein RGA3	ERO13_D11G336 100v2/chromosome D11	R-gene-mediated resistance and disease response	Chen et al. (2015)
25	107916180	LOC107 916180	Bidirectional sugar transporter SWEET17	ERO13_D10G171 600v2/chromosome D10	Confer’s tolerance against abiotic stress, pathogenesis-related protein	Chandran (2015)
26	105792917	LOC105 792917	Protein ECERIFERU M 16	B456_004G28270 0/chromosome 8	Role in biotic and abiotic stresses	Bourdenx et al. (2011)
27	107928876	LOC107 928876	Aquaporin PIP2-7-like	ERO13_D01G001 900v2/chromosome D01	Critical in plant immunity, biotic and abiotic stresses	Matsumoto et al. (2009), Meng et al. (2016), Tian et al. (2016)
28	107927933	LOC107 927933	Potassium channel AKT2/3	ERO13_A03G193 300v2/chromosome A03	Plant development, stress responses, antiviral defense	(Wang and Wu 2010, Zhou et al. 2014)
29	107937358	LOC107 937358	Boron transporter 4	ERO13_A06G187 400v2/chromosome A06	Involved in R-gene-mediated viral defense	Lim et al. (2014)
30	107949718	LOC107 949718	Vignain	ERO13_D03G102 700v2/chromosome D03	Plant immunity, pathogenesis, and plant defense	Figueiredo et al. (2014)
31	SAMS	GhSAMS	S-adenosylmethionine synthase	Chromosome AD1	Drought tolerance	Sun et al. (2022)
32	TR104989_c0_g1	LSM14-like protein B	Zf-C2H2 transcription factors	—	CLCuV resistance	Farooq et al. (2022)
33	AT5G11270.1	Gh_D08G2462	A homeodomain transcription factor	Chromosome _D8	Potential candidate gene for drought tolerance	Guo et al. (2022)
34	AT2G29560.1	Gh_A13G1963	A putative Phosphoenolpyruvate enolase	Chromosome A13	Potential candidate gene for drought tolerance	(Guo et al. 2022)
35	—	GhCFIm25	Pre-mRNA cleavage factor Im 25 kDa subunit	—	A potential candidate gene for Aphis gossypii tolerance	Yang et al. (2023)
36	—	GhGUX5	Involved in several physiological processes		A candidate gene associated with resistance to *Verticillium wilt*	Zhang et al. (2023)

### 4.1 Pathogen derived resistance strategies

RNA interference (RNAi) is another potent defense mechanism in plants. This mechanism involves the degradation of viral mRNA, effectively silencing the genes responsible for viral replication. For example, after the initial cloning of the first effector glucose oxidase from *Helicoverpa zea* (Musser et al., 2002), many other effectors like HARP1 (Chen et al., 2019), and cytochrome P450 monooxygenases (TAO et al., 2012), were discovered. In addition to phytochemicals, phytohormones (SA and JA) also have the capability to activate insect P450 genes when insects invade (Li et al., 2002). Both alterations in the structure of P450 proteins and increases in the expression of P450 genes have been linked to the ability of insects to tolerate gossypol and insecticides such as fenvalerate and deltamethrin (Joußen et al., 2012). Upon introducing a double-stranded RNA (dsRNA) construct that targeted the gossypol-inducible P450 gene *CYP6AE14* (referred to as *dsCYP6AE14*) into *Arabidopsis* plants, there was a significant reduction in the expression of *CYP6AE14*, resulting in a simultaneous decrease in the weight gain of larvae (Mao et al., 2007). Similarly, employing dsRNA constructs to target genes like *dsFAR* (fatty acyl-CoA reductase), *dsJHBP* (JH-binding protein), *dsJHAMT* (juvenile hormone acid methyltransferase) and *dsNDUFV2* (encoding a mitochondrial complex I subunit) in the sap-sucking pest *Adelphocoris suturalis* of the *Miridae* family and *Helicoverpa armigera* yielded positive outcomes when transformed into cotton plants for pest control (Huang et al., 2021). Additionally, plants produce natural defense chemicals like nitric oxide (NO), salicylic acid (C₇H₆O₃), and reactive oxygen species (ROS) to combat pathogens (Ibrahim et al., 2023). Short interfering RNAs (siRNAs) also play a role in plant defense against double-stranded RNA, although some viruses have developed proteins to counteract this response. In the event of injury, plants restrict viral movement through plasmodesmata. These findings now urge cotton breeders to use RNAi extensively in controlling CLCuD.

The primary approach to controlling CLCuD involves managing the whitefly population through insecticides. However, this method is not entirely reliable, as delayed application could lead to transmission of the infectious strain. Moreover, insecticide use has adverse environmental effects and can be toxic to various organisms. Therefore, conventional methods of protecting cotton crops from insects are not preferable. Biotechnological and molecular breeding approaches present the most promising option for inducing resistance in cotton against viruses (Saakre et al., 2023). Pathogen-derived resistance can be further categorized into two types: resistance with protein expression and resistance without protein expression. Proteins play a crucial role in plant defense against biotic stresses, with research indicating that proteins act as the primary defense against CLCuD in cotton (Chauhan et al., 2023). Viral replication, regulated by Rep proteins, can be disrupted to inhibit the synthesis of viral components. However, while efforts have been made to develop transgenic cotton varieties against CLCuD, these varieties are still undergoing testing. The second approach, without protein expression, involves inducing resistance through RNAi. RNAi is a sequence-specific defense mechanism that leads to the degradation of viral mRNA transcripts. In plants, RNAi can be triggered by introducing a hairpin loop structure with a sequence homologous to the virus, thus stimulating the silencing signal (Karthik et al., 2023). This approach could also aid in developing insect-resistant genes against whitefly-transmitted viruses. RNAi-based resistance has been successful against Bean Golden Mosaic Virus in beans in Brazil (Souza et al., 2018). Another approach involves coat protein (CP) expression from other viruses, such as the Tobacco Mosaic Virus (TMV), to induce resistance. For example; Rasool et al. (2021), in their study, used synthetic genes to explore pathogen-derived resistance (PDR) for managing CLCuKoV-Bu and its associated betasatellite linked to CLCuD. Synthetic coat protein (CP_syn_) and replication-associated protein (Rep_syn_) genes from CLCuKoV-Bu were designed and inserted into *Nicotiana benthamiana* plants via *Agrobacterium*-mediated transformation. Transgenic plants carrying CP_syn_ and Rep_syn_ genes showed milder symptoms and lower virus concentrations when exposed to CLCuKoV-Bu or CLCuKoV-Bu-CLCuMuB inoculums. These findings suggest that CP_syn_ and Rep_syn_ genes have the potential for conferring resistance against CLCuKoV-Bu and related betasatellites and the study indicates the development of a PDR mechanism for controlling CLCuD. However, the exact mechanisms behind these strategies remain only partially understood (Rasool et al., 2021).

### 4.2 Non-pathogen derived resistance strategies

Non-pathogen-derived resistance strategies have been explored in cotton against CLCuD. The Need for non-pathogen-derived resistance strategies emerged when the applications of biotechnological approaches were found successful in other crops like wheat (Jastrzębska et al., 2020), rice (Montesinos et al., 2017), and maize (Gould and Northcote, 1986), but with a low success ratio in cotton (Khan et al., 2015). These strategies include enhancing natural defenses like trichomes (which are hair-like structures, developed at the aerial plant surfaces involved in plant defense) and increasing the concentration of waxes and inorganic salts, which can protect cotton plants against whiteflies, CLCuD, and other pests. For example; Suthar et al., 2021, studied the tolerance response of interspecific derivatives (resulting from a cross between *Gossypium hirsutum* L. × *G. armourianum Kearney*) against whitefly. Their results revealed that the concentration of trichome at early developing stages was high and developed strong tolerance against whitefly. However, with age progression, a significant decline was observed in trichome concentration in mature leaves (Suthar et al., 2021). Majid et al. (2020) conducted an experiment to study the effect of epicuticular wax against CLCuD in different varieties of cotton crop. Their results revealed a strong significant negative correlation between leaf epicuticular wax and CLCuD infection, indicating that the higher the concentration of wax the lower will be the CLCuD infection (Majid et al., 2020).

## 5 Revolutionizing genetics: genome editing and beyond

### 5.1 CRISPR-Cas mediated genome editing for CLCuD

Genome editing (GE) technologies have brought about a transformative shift in the realm of plant research and hold immense promise for enhancing crop attributes. The Clustered Regularly Interspaced Short Palindromic Repeat (CRISPR)-CRISPR-associated protein (CRISPR-Cas) system, renowned for its adaptability, simplicity, and cost-effectiveness in achieving precise modifications to DNA sequences such as gene knockout or knockdown, allele/gene *in vivo* replacement, and single base substitution, has emerged as the dominant force in GE over recent years (Khan et al., 2022; Atiq et al., 2023). Upon creating double-strand DNA breaks (DSBs) by CRISPR-Cas, subsequent repair occurs via either the error-prone non-homologous end joining (NHEJ) pathway or the more precise homology-directed repair (HDR), or a combination of both pathways (Liu et al., 2022). NHEJ primarily facilitates DSB repair and typically induces random indels (also called insertions and deletions) at the rejoining point of the chromosomes. This propensity for indels has led to the predominant use of NHEJ in current plant GE endeavors for inducing gene knockouts and mutations. In the context of precise genome editing, HDR can be invoked through the presence of a DNA or RNA donor repair template (DRT) containing homologous sequences encircling the DSB. This permits more precise gene insertions or replacements (Tang et al., 2023). Alternatively, two other strategies also exist that still offer precise GE even without DRTs and DSBs. The first is called base editing (BE), a powerful technique facilitating gene replacement through HDR, enabling the precise substitution of a single base with a designated single nucleotide polymorphism (SNP) (Sony et al., 2023). This technique facilitates the precise alteration of plant genomes in a programmed manner. The second strategy, known as prime editing (PE), greatly extends the scope of precise genome editing by allowing all types of minor indels and base substitutions (Karlson et al., 2021). Near in the future, the widespread adoption of various CRISPR toolsets will significantly hasten progress in both crop genetic improvement and biological research, particularly for those plant species with available reference genome sequences.

Until now, most of the research on genome editing in crop plants, such as cotton, has reliedon NHEJ to create targeted loss-of-function mutations at particular gene locations. The applications of CRISPR-Cas9 have been widely employed to enhance both crop yield and quality such as resilience against abiotic and biotic factors including CLCuD. For instance; Binyameen et al. (2021), used multiplexed CRISPR-based GE system to develop resistance against CLCuD in cotton. They obtained the targeted CLCuD sequences from the National Center for Biotechnology Information (NCBI) and designed three guided RNA (gRNA) molecules to target three different sites in the genome. They successfully obtained 60%–70% resistance against CLCuD at the seedling stage compared with control plants (Binyameen et al., 2021). The βC1 and Rep genes were responsible for replication of CLCuV within the host genome. Using a newly developed multiplexed GE approach was more efficient and effective to knock down or knock-out these genes to develop resistance against CLCuD (Khan et al., 2020). Improving cotton quality and meeting the needs of the cotton industry is an important breeding objective for cotton breeders. The aim is to develop a cotton variety that not only showcases improved quality and resistance against CLCuD but also achieves increased production without sacrificing yield.

### 5.2 Base editing and its applications against CLCuD

BE offers a mechanism to achieve exact substitutions of one base pair for another at a designated genomic position, without necessitating a DRT and without instigating DSBs (Basu et al., 2023). This technique operates independently of the HDR pathway. Recently existing base editors, encompassing adenine base editors (ABEs) and cytosine base editors (CBEs), have been engineered by fusing the inactive Cas9 (dCas9) or Cas9 nickase (D10A) (nCas9) with adenine deaminase or cytidine deaminase, respectively (Komor et al., 2016; Gaudelli et al., 2017). In this manner, single-point mutations such as the conversion of A*T to G*C or C*G to T*A in crop plants can be generated using ABEs or CBEs (Bharat et al., 2020; Ali et al., 2023; Khan et al., 2023). So far, base editing has been effectively used to target several important traits in various crops like maize, cotton, *Arabidopsis thaliana*, rice and others. For example,; Qin et al. (2020), developed the novel *G. hirsutum*-Base Editor 3 (GhBE3) to induce single base mutation in the allotetraploid genome of G. *hirsutum*. To test the efficiency of this system, two genes (*GhPEBP* and *GhCLA*) were targeted at three different sites in the genome. The efficiency of this base editing system was found highly effective and ranged from 26.67% to 57.78% (Qin et al., 2020). In another study, Wang et al. (2022), created diverse ABE vectors utilizing modified adenosine deaminase (TadA) proteins linked with two Cas9 variants named nCas9 and dCas9. These vectors demonstrated proficient A to G editing, achieving efficiencies up to 64% at targeted locations in the complex allotetraploid cotton genome. The results revealed *GhABE7.10n* as the most effective, particularly altering the A5 position (considering PAM as positions 21–23). RNA off-target and DNA assessments using *GhABE7.10d* and *GhABE7.10n* edited cotton plants revealed minimal RNA off-target mutations and no DNA off-target changes. Additionally, a novel base editor, *GhABE7.10d*Cpf1, was introduced, recognizing a PAM rich in thymine (T), leading to targeted A-to- G substitutions and a single amino acid modification in the cotton phosphatidyl ethanolamine-binding protein (*GhPEBP*). This alteration resulted in a condensed cotton plant structure that is ideal for mechanized harvesting in contemporary cotton farming (Wang et al., 2022). Recently, Mubarik et al. (2021) demonstrated a successful CRISPR-Cas9 system application to combat CLCuD by targeting overlapping genes of prevalent CLCuVs with three guide RNAs (gRNAs). Utilizing a multiplex CRISPR-Cas9 construct targeting six CLCuV genes proved more effective in inhibiting virus proliferation compared to individual targeting. Targeting CLCuV at multiple sites simultaneously resulted in superior interference and inefficient recovery of altered virus molecules. When tested in cotton plants, the multiplex construct significantly reduced virus accumulation in leaves. These findings highlight the potential of CRISPR-Cas9 for engineering virus resistance in crops, especially against mixed virus infections, through multiplex genome editing.

### 5.3 Prime editing and its applications against CLCuD

Prime editing, also known as PE, is a revolutionary genome-editing approach that allows precise modifications to the genetic code. Unlike traditional methods that rely on donor DNA templates or DSBs, prime editing utilizes a fusion protein complex to introduce specific edits at desired gene loci in plant cells (Hillary and Ceasar, 2022). Prime editing involves the conjugation of a reverse transcriptase Moloney murine leukemia virus reverse transcriptase (M-MLV-RT) with a catalytically impaired Cas9 (nCas9) to form a protein complex. A guide RNA for prime editing (pegRNA) is employed to designate the desired target location and encode the intended genetic information. Within the prime editing system, the protein complex attaches to the targeted DNA sequence and creates a nick in the non-target strand (Li et al., 2021). Prime editing has gone through several iterations, with each version aiming to improve product purity and editing efficiency. The initial version, PE1, was developed by fusing the C-terminus of nCas9 withM-MLV-RT. This fusion protein showed promise but lacked optimal efficacy. To improve PE efficacy, PE2 was developed by replacing the original M-MLV-RT with an engineered version containing six mutations: *L603W*, *T330P*, *W313F*, *T306K*, *D200N*, and *H9Y*. Building upon the success of PE2, PE3 was created by introducing another nicking sgRNA at various distances from the nicks induced by the pegRNA. This additional cleavage in the non-edited strand further increased the efficiency of the editing process (Anzalone et al., 2019).

PE shows significant potential for introducing precise genome modifications in cotton crop to enhance agronomic traits and develop resistance against CLCuD. In cotton, CLCuD tolerant plants were developed using prime editing (Cao et al., 2020). The multiple gRNA was cloned to target C1 and Rep genes simultaneously. The resulted cottons plants did not display any CLCuD symptoms after 30 days of transformation and the results were further confirmed through PCR by extracting the RNA at different growth stages (Shafique et al., 2022). Shortly, prime editing represents a significant advancement in genome-editing technology, offering improved precision and efficiency compared to traditional methods (Lin et al., 2020). The development of various prime editing systems, from PE1 to PE3b, has enabled researchers to achieve precise modifications in both human cells and crop plants. As the technology continues to evolve, prime editing may become a powerful tool in the hands of cotton breeders for creating sustainable and resilient cotton varieties to meet the challenges of the future (Li et al., 2020).

## 6 Beyond genome editing

### 6.1 Next-generation sequencing technologies

Cotton has been extensively studied in the field of genomics to understand its genetic makeup and improve its agronomic traits. In the past, classical methods such as microarray analysis were used to study the genetics of plants, but they were costly, low-yielding, slow and labor-intensive (Hu et al., 2021). However, in the last two decades, high-throughput sequencing methods, collectively called Next-Generation Sequencing (NGS), have revolutionized plant genetics research, including cotton. NGS methods offer higher throughput, faster turnaround times, and reduced costs compared to traditional sequencing methods (Salk et al., 2018).

### 6.2 First generation technology

The first generation of sequencing technology, which emerged in the early 2000s, included chemical analysis and enzymatic sequencing methods. These methods relied on chain termination using 2′,3′- dideoxynucleotides (ddNTPs) (Medžiūnė et al., 2022). First-generation sequencers, such as the ABI Prism, were developed to automate the sequencing process and could analyze 4 to 96 samples per single run (Kchouk et al., 2017). Although this technology produced high-quality sequencing, it had limitations in terms of laboriousness, slow methodology, and limited data output. It was primarily used for small genomes and large sequencing projects like the Human Genome Project (Collins et al., 2003). Mansoor et al. (1999) identified and characterized a new circular single-stranded (ss) DNA found in naturally infected cotton plants. Purified geminate particles from infected plants not only contain a component resembling DNA A but also a smaller, unrelated ssDNA termed DNA 1. DNA 1 was cloned from the double-stranded replicative form of viral DNA isolated from infected cotton plants. Blot hybridization using specific probes for CLCuV DNA or DNA 1 demonstrated the co-infection of naturally infected cotton plants in various geographical locations by both DNAs. DNA 1 was detected in viruliferous Bemisia tabaci and in laboratory-infected tobacco plants using B. *tabaci*, indicating its transmission by whiteflies. Sequence analysis revealed that DNA 1 is approximately half the size of CLCuV DNA, with no homology, suggesting it is not a defective *Geminivirus* component. DNA 1 shares some homology with a genomic component of Nanoviridae, a family of DNA viruses typically transmitted by aphids or planthoppers. DNA 1 encodes a homologue of the nanovirus replication-associated protein (Rep) and can autonomously replicate in tobacco. The findings imply that a nanovirus-like DNA has acquired whitefly transmissibility through its association with a *Geminivirus*. This association may contribute to a mutually dependent relationship between two distinct DNA virus families with a similar replication strategy, potentially influencing the development of cotton leaf curl disease. Similarly, Sharma et al. (2005) used polymerase chain reaction (PCR) technique to detect viral DNA in samples stored for up to 3 days after collection, making it widely applicable for field diagnosis. In their study, the complete nucleotide sequence of the coat protein (CP) gene component of the Indian isolate of cotton leaf curl *Geminivirus* (CLCuV-HS2) was determined using CP-specific primers in PCR amplification. The infected cotton plants were obtained from fields in Haryana, India. Amino acid sequence comparison of the putative CP with other mono and bipartite *Geminiviruses* revealed a maximum identity of 97.3% with Pakistan cotton leaf curl virus (CLCuV-62). Additionally, a nuclear localization signal close to the N-terminal of the CP gene was identified. Moreover, Briddon et al. (2000) obtained full-length clones of Cotton Leaf Curl Virus (CLCuV), equivalent to the DNA A component of bipartite *Begomoviruses*. These CLCuV clones were found to be systemically infectious in both Nicotiana benthamiana and cotton plants. Interestingly, the infected plants did not display the typical symptoms associated with cotton leaf curl disease; instead, they exhibited mild leaf curling, yellowing, and some stunting. Despite efforts to identify a second genomic component, none was found. These results suggest that CLCuV alone may not be the sole cause of cotton leaf curl disease. However, the transmission of the disease by Bemisia tabaci, a whitefly, implies that the *Begomovirus* CLCuV might still play a role in disease transmission, even if it is not the primary causative agent.

### 6.3 Second generation technology

The advent of second-generation sequencing technology marked a significant milestone in the field of genomics. Unlike first-generation methods, second-generation or short-read sequencing techniques utilized PCR to amplify DNA fragments before sequencing. These techniques employed hybridization or synthesis-based methods to sequence millions of DNA fragments in parallel, enabling high-throughput sequencing (Adewale, 2020). Platforms like 454 Life Sciences Roche, SOLiD (Thermo-Fisher Scientific), Solexa (later acquired by Illumina), and Ion Torrent revolutionized the sequencing landscape. Illumina, the extensively employed technology for genotyping investigations in cotton, provides diverse choices for transcriptome and genome sequencing, delivering outputs spanning from gigabases to terabases (Meera Krishna et al., 2019). Patel (2016), detailed the *de novo* assembly process and characterization of transcriptome and genome data for an Asia I population. The research aimed to establish a genetic framework for insecticide discovery. Using the Roche 454 sequencing platform, the authors assembled a comprehensive transcriptome of adult females from the Asia I population, generating 0.864 million reads and producing 29,418 contigs through CLC Genomics. Of these, 8,563 contigs were assigned putative functions. A draft genome of 828 Mbp for the Asia I population was constructed via *de novo* assembly of 206 million 250 bp paired-end reads from the Illumina MiSeq 2,500 platform using Platanus. This genome comprised 41,981 protein-coding genes (PCGs) and included 990 non-coding RNAs and repetitive elements (45.66%). The study examined 741 full-length genes, revealing larger intron sizes and repetitive elements contributing to the larger Asia I genome size, which explained challenges in genome assembly. Additionally, the research obtained one mitogenome and three endosymbiont genomes (Portiera, Wolbachia, and Arsenophonus) from the same sequence library, alongside the B. *tabaci* Asia I genome. The development of a genetic framework involved using *Drosophila* essential genes as a reference to identify orthologs in other insects, validated with ChEMBL targets. The presented ‘omics’ data serves as a comprehensive sequence resource for Asia I populations, illustrating the workflow for obtaining genetic information on hosts and their endosymbionts.

### 6.4 Third generation technology

Third-generation sequencing techniques, often referred to as single molecule sequencing (SMS), represent the latest advancement in sequencing technology. These methods do not require PCR amplification and can sequence single DNA molecules directly. The ability to sequence DNA molecules without amplification provide advantages such as reduced bias, improved accuracy, and the ability to detect modified bases. PacBio’s Single Molecule Real-Time (SMRT) sequencing (Ardui et al., 2018), and Oxford Nanopore Technologies’ nanopore sequencing (Weirather et al., 2017) are two prominent examples of third-generation sequencing platforms. These technologies have opened up new avenues for studying viral diversity, metagenomics, and epigenomics. Vij et al. (2022) presented a groundbreaking study on the introgression and mapping of Cotton Leaf Curl Disease (CLCuD) resistance from a ‘synthetic cotton polyploid’ to upland cotton. They developed a backcross population (synthetic polyploid/Gossypium hirsutum Acc. PIL 43/G. hirsutum Acc. PIL 43) to investigate the inheritance and mapping of CLCuD resistance. The study observed the dominance of CLCuD resistance over susceptibility, revealing the presence of two dominant genes conferring resistance to CLCuD. Molecular analysis using genotyping-by-sequencing identified chromosomes A01 and D07 as carrying one CLCuD resistance gene each. This research marks the first instance of successfully transferring and mapping CLCuD resistance from a synthetic polyploid to upland cotton. Furthermore, Hussain et al. (2023) conducted a sequencing study on the Mac7 accession, a source of Gossypium hirsutum known for its resistance against various biotic stresses. Through alignment with the Gossypium hirsutum ‘TM-1′ genome (AD1), they identified 4.7 million SNPs and 1.2 million InDels in the Mac7 genome. Gene ontology and metabolic pathway enrichment analyses indicated the involvement of these SNPs and InDels in processes such as nucleotide binding, secondary metabolite synthesis, and plant-pathogen interaction pathways. Additionally, RNA-seq data from different tissues and qPCR expression profiling under Cotton Leaf Curl Disease (CLCuD) revealed the roles of individual genes in resistant and susceptible accessions. Notably, the expression of differential NLR genes was higher in resistant plants compared to susceptible ones. The resequencing results offer foundational data for identifying DNA resistance markers, potentially aiding marker-assisted breeding for the development of Mac7-derived resistance lines. Ahmed et al. (2021), utilized CIDER-seq (Circular DNA Enrichment Sequencing), a recently developed PCR-free virus enrichment sequencing method, in conjunction with Sanger sequencing to investigate the genetic diversity of the Cotton leaf curl disease (CLCuD) complex. The study identified a highly recombinant strain of Cotton leaf curl Multan virus and a recently evolved strain of Cotton leaf curl Multan betasatellite, both prevalent in major cotton-growing regions. Additionally, multiple species of alphasatellites were identified, including Mesta yellow vein mosaic alphasatellite (MeYVMA) observed for the first time in cotton. The study also employed real-time quantitative PCR to determine the relative abundance of the virus and associated satellites. This research represents the first study to characterize the CLCuD complex during its third epidemic.

## 7 Next-generation sequencing applications in cotton genomics

### 7.1 Genome sequencing and assembly

NGS technologies have displayed a significant role in sequencing and assembling the cotton genome. The availability of the cotton sequenced genome has facilitated the discoveries of genes responsible for important cotton traits, like disease resistance (CLCuD) and fiber quality. Both diploid and tetraploid cotton genomes have been sequenced using NGS technologies, providing valuable insights into the genetic basis of cotton traits. For example,; Hussain et al. (2023), sequenced

*G. hirsutum* accessions named Mac7 (known to possess resistance against several biotic stresses) to identified the genetic bases associated with resistance against CLCuD. A total of 1.2 and 4.7 million InDels and SNPs were identified and their roles in resistance against CLCuD were accessed using metabolic pathway enrichment and gene ontology. Furthermore, RNA-seq data collected from different tissues at different growth stages identified individual genes associated with resistance and susceptibility in Mac7 against CLCuD (Hussain et al., 2023). Virus is famous for changing its strain rapidly and it became difficult to separate the newly developed strain from the previous one.

Genome sequencing technologies have now made it simple to separate the new strain one from the previous one and also detect the genes/alleles associated with the CLCuD. For example,; Kumar et al. (2010) collected unknown leaf sample affected with CLCuD from two different sites of Rajasthan and DNA-A components of both samples were characterized and amplified. DNA-A of one sample consist of 2,759 nucleotides and the second sample consist of 2,751 nucleotides with both having total of six open reading frames (ORF’s). Upon sequence characterization, the first sample showed sequence similarity with CLCuV strain reported in Pakistan and the second sample showed sequence similarity with the local Rajasthan CLCuV strain. These sequence findings suggest a potential movement of certain strains of the CLCuV from Pakistan to India, or the concurrent existence of diverse isolates in similar geographical conditions. Whereas CLCuV-SG01 shared the highest nucleotide sequence similarity with CLCuV Rajasthan (Abohar), the V1 ORF (encoding coat protein) of SG01 displayed the highest nucleotide identity (100%) with CLCuV Multan (Bhatinda) and Abohar virus, albeit with variations in the AC1 region. The entire nucleotide sequence of SG01 exhibited an 86% similarity to CLCuV Multan virus. A similarity search unveiled significant disparities in AC1 and AV1 regions concerning DNA-A, indicative of a recombination-driven evolutionary history. Computational analysis through the Recombination Detection Program (RDP) supports this recombination hypothesis, suggesting the occurrence of recombination with other *Begomoviruses* within the AC1-ORF of CLCuV-SG01 and V1 ORF, as well as the AC1-ORF of CLCuV-SG02, alongside the intergenic noncoding region (Kumar et al., 2010).

### 7.2 Transcriptome analysis and gene expression profiling

RNA sequencing (RNA-seq) has become a popular approach for studying gene expression in cotton. By sequencing the transcriptome, researchers can identify differentially expressed genes under various conditions, such as stress, development, and tissue-specific expression (Berkowitz et al., 2021). RNA-seq has enabled the discovery of non-coding RNAs, alternative splicing events and novel transcripts in cotton (Qin et al., 2022; Singh et al., 2023). The commonly grown cotton species, *Gossypium hirsutum* is easily affected by CLCuD whereas; the diploid species *G. arboreum* naturally has some resistance (Hussain et al., 2023). However, the mechanism behind how CLCuD affects the genes expression profile of *G. arboreum* and how the disease interacts with it is unclear. Therefore, Naqvi et al. (2017) utilized RNA-Seq technique to look at how genes behave in *G. arboreum* when it is dealing with CLCuD. They infected *G. arboreum* plants with CLCuD by 600 grafting parts from infected *G. hirsutum* plants and studied the genes in both healthy and infected *G. arboreum* plants using RNA-seq with an Illumina HiSeq 2,500 machine. A total of 1,062 differentially expressed genes (DEGs) were identified of which 17 significant DEGs having possible role in disease resistance were studied for their expression profiles using qPCR. They also found genes that are related to fighting the disease and protecting the plant. Using the RNA-Seq data, they made a network of genes that work together. Within this network, they identified 50 hub genes, many of which help transport things inside the plant. These genes might be important for how *G. arboreum* defends itself against CLCuD. This study is a starting point for understanding how the virus and the plant interact and finding the key genes that help *G. arboreum* resist CLCuD (Naqvi et al., 2017). Furthermore, transcriptome analysis and gene expression profiling offer insights into how the plant reacts when CLCuD is transmitted through whiteflies in *G. hirsutum*. Naqvi et al. (2019) utilized RNA-Seq approach to delve into the variations in gene activity within the susceptible *G. hirsutum* variety when it is infected by whiteflies carrying the CLCuD. To compare, they subjected both uninfected and CLCuD-infected plants to RNA-Seq analysis using the Illumina HiSeq 2,500 platform. They identified 468 differentially expressed genes (DEGs), consisting of 248 downregulated and 220 upregulated DEGs that play roles in responding to diseases and defending against pathogens. Ten of these genes were selected for further analysis using RT-qPCR on two susceptible cultivars, MNH 786 and Karishma. The consistent gene expression patterns across both cultivars affirmed the reliability of their transcriptome data, suggesting the applicability of their study’s insights into the broader context of host susceptibility to CLCuD. They proceeded to conduct weighted gene co-expression network analysis, revealing six modules within the gene interaction networks. This analysis also pinpointed highly co-expressed genes, with 55 hub genes demonstrating co-expression with at least 50 other genes. Notably, many of these hub genes were found to be downregulated and involved in cellular processes. The diminished expression of these co-expressed genes indicated their potential role in favoring the virus and amplifying the plant’s vulnerability to CLCuD. They also discuss the potential mechanisms underpinning the establishment of disease susceptibility. Altogether, their study presents a comprehensive analysis of gene activity changes in *G. hirsutum* when infected by whitefly transmitted CLCuD. This research contributes to a better understanding of the combined impact of whiteflies and the virus on their host, offering insights into significant *G. hirsutum* genes that play a role in its susceptibility to CLCuD (Naqvi et al., 2019). Transcriptome analysis and gene expression profiling have also been successfully applied to crops other than cotton, such as wheat (Lee et al., 2022; Xu et al., 2022), rice (Thomas et al., 2019; Kong et al., 2020), maize (Wang et al., 2019; Zhang et al., 2020), tomato (Wen et al., 2019; Riccini et al., 2021) and many others.

### 7.3 Identification of genetic variation

The creation of genetic diversity has always been the prime target of plant breeders to improve crop growth, development, and yield. Plant breeders use different techniques from hybridization to induce mutations (chemical or physical) to create genetic diversity in plants (Wang et al., 2020). In the case of CLCuD, inducing mutation via chemical or physical means to develop resistance against CLCuD in susceptible cotton genotypes is another modern breeding technique (Rehman et al., 2023). However, after inducing tolerance against CLCuD via mutation in susceptible line, detecting the mutated gene or single nucleotide polymorphisms (SNPs) associated with resistance is another objective of breeders which can only be detected after whole genome sequencing (Chen et al., 2022). NGS technologies have revolutionized the identification and characterization of genetic variation in cotton. By sequencing multiple cotton genotypes, researchers can detect SNPs, insertions, deletions, and structural variations. This information is crucial for marker-assisted selection (MAS) or marker-assisted breeding (MAB), genetic mapping, and understanding the complex traits genetic architecture in cotton (Kushanov et al., 2021). Molecular markers have shown important role in agriculture for cultivar and agronomical trait improvement such as detection of resistance genes in plants. Numerous QTLs have been developed based on different experimental designs and genome mapping has been greatly improved and has provided a number of QTLs for major and minor crops including cotton (Zargar et al., 2015). Molecular markers linked with QTLs have been identified for various diseases in different crops. MAS has been successfully used in the development of resistant varieties. In cotton, QTLs related to disease are described as simple inherited traits. However, molecular breeding for disease resistance/tolerance is more specific, that is why a lot of work is needed to be done for locating QTLs linked with CLCuD resistance in cotton (Javed et al., 2019). In the recent years, genome map construction has gained an immense significance in MAS. MAS has been acquired as a molecular tool for the selection of desirable phenotypes using DNA markers such as RFLP, RAPD and AFLP on the basis of agriculturally important traits and the genes linked with tolerance against several abiotic and biotic factors (Jones et al., 1997; Khan et al., 2023). Use of MAS in plant breeding has opened new doors in crop improvement (Winter and Kahl, 1995).

Although, CLCuD has always been an important topic of discussion for botanists, yet exact opinion is still needed to be developed on the inheritance and spread of CLCuD (Mahmood et al., 2003). A study conducted by Rahman et al. was based on the collection of minor genes associated with CLCuV disease resistant material, selected by recurrent method (Rahman et al., 2005). Resistance is controlled by major genes, which are dominant and may be affected by the evolution of pathogens (Iqbal et al., 2003).

### 7.4 Epigenomics and DNA methylation analysis

Epigenetic modifications, such as DNA methylation, play a crucial role in regulating gene expression. NGS technologies have enabled the study of DNA methylation patterns in cotton, providing insights into epigenetic regulation of important agronomic traits. DNA methylation analysis can help understand the impact of environmental factors on gene expression and phenotype (He et al., 2022). DNA methylation in plants take place at cytosine residues in the context of CHH, CHG and CG (where H can be T, C or A) sequences (Feng et al., 2020). DNA methylation patterns can be heritable and can influence gene expression and phenotype. In cotton, DNA methylation has been shown to play a role in various developmental processes, stress responses, and disease resistance. To discover DNA methylation alterations during cotton domestication and evolution, researchers have developed single-base resolution methylomes from different cotton species and varieties. They found significant differences in DNA methylation patterns among diploid and tetraploid cottons, as well as between wild and cultivated cottons (Song et al., 2017). These DNA methylation changes were found to be associated with gene expression changes and may have contributed to the domestication and adaptation of cotton plants. Moreover, transposable elements (TEs) are usually linked with DNA methylation and can contribute to complexity of genome. In diploid cotton species, there are differences in TE content and DNA methylation patterns between different species (Song et al., 2017). The presence of TEs in genic regions can affect gene expression and evolution. The study of DNA methylation divergence among diploid cotton species provides deep information in understanding the role of DNA methylation in genome evolution and speciation. DNA methylation can play a role in disease resistance in plants (Tirnaz and Batley, 2019). In cotton, DNA methylation changes have been associated with resistance to CLCuD. The betasatellite, a key component of the CLCuD complex, is involved in manipulating host cellular functions and suppressing gene silencing. DNA methylation changes in response to betasatellite infection may contribute to disease resistance by regulating gene expression and host defense responses. qPCR analysis has been used to evaluate the CLCuD-associated *Begomoviruses* titers and their impact on cotton yield. qPCR is a powerful method for quantifying the levels of virus and satellite DNA in infected plants. The results of qPCR analysis have unveiled a significant negative correlation between betasatellite titer and seed cotton yield (SCY) (Iqbal et al., 2023). This finding suggests that betasatellite titer is a critical factor in determining cotton yield and productivity.

### 7.5 Functional genomics

Functional genomics has emerged as a crucial scientific discipline in recent years, revolutionizing our understanding of plant biology and genetics improvement (Van Bel et al., 2022). In the case of cotton, functional genomics offers the potential to systematically exploit genetic resources, improve resistance against biotic stresses, enhance yield, and optimize germplasm for cultivation (Chen et al., 2007; Wei et al., 2022). However, several challenges persist in cotton functional genomics, including the long growth cycle, low transformation efficiency, large genome size and the lack of efficient molecular tools. Nonetheless, considerable advancements have been achieved in the development of genetically modified cotton varieties, particularly against resistance to CLCuD and other insects (Shafique et al., 2022). The sequencing of genomes plays a pivotal role in functional genomics. In 2007, the Cotton Genome Consortium strategized to sequence cotton genomes, prioritizing less-complicated diploid genomes that could be directly applied to tetraploid cotton (Wang et al., 2012). Consequently, the draft genome sequence of *G. raimondii*, a diploid species, was released in 2012, providing valuable insights into the larger ‘A' diploid and ‘AD’ tetraploid cotton genomes (Li et al., 2014). Subsequently, the genome of *G. arboreum*, a supposed donor species for the A chromosome group in tetraploid cotton, was also sequenced (Watson et al., 2018). However, the precise species directing the development of tetraploid cotton currently remains unknown, highlighting the need for further genome sequencing to understand the biology and evolutionary history of CLCuD. Despite the availability of reference genome sequences for tetraploid and diploid cotton species, some discrepancies exist, potentially due to assembly errors. Therefore, efforts should be directed towards improving the quality control standards for genome assemblies and re-sequencing for accurate comparison and analysis of different cotton species. Vij et al. (2022) used one of the functional genomics approach (genotyping by sequencing) to detect the genes associated with the CLCuD in both upland cotton and backcross population (synthetic cotton polyploidy). They identified two potential genes on chromosomes D07 and A01 that confer resistance to CLCuD (Vij et al., 2022). Similarly; Farooq et al. (2022), compared the CLCuD transmission behavior of different whitefly species (MEAM1 and Asia II 7) with different time frames using transcriptomics. Sequencing using Illumina technology unveiled, after 6 and 12 h of acquiring CLCuD, different sets of genes were differentially expressed in viruliferous (VF) whiteflies (MEAM 1 and Asia II 7) compared to aviruliferous (AVF) whiteflies. The main categories of these DEGs were associated with transcription factors (TFs) of HTH-1 class in Asia II 7 and zf-C2H2 class in MEAM1. Interestingly, MEAM1 species showed more transcriptional changes and increased immune-related responses after CLCuD infection compared with Asia II 7. Both species had specific genes linked to antiviral innate immunity, lysosome function, autophagy/phagosome pathways, spliceosome, detoxification, signaling pathways, carbohydrate metabolism, transport, and protein digestion. Validation through RT-qPCR and RNA-seq showed consistent expression in 23 out of 28 selected genes. These findings provide insights into how *Begomoviruses* interact with whiteflies, affecting their transmission dynamics, and offer new targets for managing insect-transmitted plant viruses in a sustainable way (Farooq et al., 2022).

To facilitate the comprehensive study of cotton genomes, several functional genomics databases have been developed for the cotton research community. These databases provide access to crucial information such as genome sequences, map positions, protein expression and mRNA data, allelic variation and metabolic pathways. CottonGen (https://www.cottongen.org), is a curated web based intellectual database that offers convenient access to cotton genetic and genomic data. It includes germplasm resources, genes, trait loci, markers, genetic maps, unigenes from ESTs and annotated whole-genome sequences. Another significant database, Cotton Functional Genomic Database (CottonFGD; https://cottonfgd.org), provides access to transcriptome data, functional annotations, genome sequences, and genome re-sequencing data for sequenced *Gossypium* genomes. Additionally, databases like the Database for Co-expression Networks with Function Modules (ccNET; http://structuralbiology.cau.edu.cn/gossypium/), Join Genome Institute (JGI; http://jgi.doe.gov), Comparative Evolutionary Genomics of Cotton (http://cottonevolution.info/), Platform of Functional Genomics Analysis in *Gossypium raimondii* (GraP; http://structuralbiology.cau.edu.cn/GraP/about.html), Cotton Genome Database (CottonDB; http://www.cottondb.org), and Cotton Genome Resource Database (CGRD; http://cgrd.hzau.edu.cn/index.php) offer valuable resources for in-depth analysis and exploration of cotton functional genomics.

### 7.6 Speed breeding

In the world of plant breeding, the traditional methods of crossbreeding and selection can be 757 time-consuming and labor-intensive. However, with the advent of speed breeding, plant breeders now have a powerful tool at their disposal to accelerate the generation of new plant varieties. Speed breeding involves manipulating environmental factors to promote rapid plant growth and shorter generation times and development of tolerance against diseases like CLCuD (Jähne et al., 2020). Plant breeding has long been a slow and meticulous process, often taking years or even decades to develop new crop varieties with desirable traits. This time-consuming nature of traditional breeding techniques has posed challenges in addressing urgent agricultural needs, such as developing crops with high nutritional profile, higher yield, and resilience to biotic and abiotic factors. This is where speed breeding comes into play (Ghosh et al., 2018).

Speed breeding offers a way to shorten the breeding cycle by creating optimal growth conditions for plants. By manipulating factors such as light exposure, temperature, and humidity, breeders can accelerate plant growth and achieve multiple generations within a single year. This rapid generation turnover opens up new possibilities for crop improvement and allows breeders to respond more effectively to emerging challenges in agriculture (Chiurugwi et al., 2019). To combat CLCuD and develop resistant cotton varieties, speed breeding has emerged as a promising technique. By accelerating the breeding cycle, breeders can quickly screen large populations of cotton plants for disease resistance and select the best-performing individuals for further breeding. This rapid selection process allows breeders to develop new cotton varieties with improved resistance to CLCuD in a shorter timeframe. So far, no research has reported the application of speed breeding against CLCuD in cotton. In addition to cotton, speed breeding has been used successfully in other cereal crops such as wheat and rice to improve disease resistance and final grain yield (Alahmad et al., 2018; Chimmili et al., 2022; Schoen et al., 2023). Given the successful use of speed breeding in other crops, cotton breeders and molecular biologists are now working on its use to improve biotic and abiotic stress tolerance in cotton.

One of the critical environmental factors in speed breeding is lighting. Plants have well-established light-sensitive receptor pathways that respond to specific wavelengths of light, triggering growth and development processes (Hickey et al., 2019). In speed breeding, breeders use specialized LED lamps to provide the necessary light spectrum for optimal plant growth and development. LED lamps offer several advantages over traditional lighting sources, such as fluorescent lamps. LED lamps can be precisely tuned to emit specific wavelengths of light; allowing breeders to customize the light spectrum based on the crop and desired traits (Gavhane et al., 2023). By manipulating the blue and redlight wavelengths, breeders can induce early flowering in plants, effectively shortening the breeding cycle. Furthermore; Nexsel, a leading provider of speed breeding solutions, has been at the forefront of developing optimized lighting protocols for various crops, including cotton. With their specialized grow cabinets and a tailored lighting solution, Nexsel offers a comprehensive package for speed breeding in cotton. The Nexsel Speed Breeding Growth Chamber provides precise control over critical parameters such as temperature, humidity, CO_2_ levels, lighting spectrum, light intensity, and photoperiod. This level of control allows breeders to create the ideal growth conditions for cotton plants, maximizing their potential for rapid growth and development. Nexsel’s lighting solutions for speed breeding, such as the All-in-One Grow Light and the 3-in-1 Grow Light, offer customizable spectra and intensity options to meet the specific needs of cotton plants at different growth stages (Jois, 2021; Gavhane et al., 2023).

## 8 Current challenges and future directions

In recent years, advanced biotechnological and molecular breeding techniques have shown promise in the field of cotton research. Precision genome editing *via* multiplex genome editing and HDR have emerged as potential strategies for controlling CLCuD and improving cotton varieties. Additionally, the *de novo* domestication of wild species and the use of synthetic biology approaches offer new avenues for developing resistance against CLCuD and enhancing cotton productivity.

### 8.1 Precision genome editing via HDR in cotton

Precision genome editing through HDR holds great potential for accelerating cotton improvement and developing resistance against CLCuD. HDR allows for the precise replacement of existing alleles, offering a more targeted and efficient approach compared to traditional breeding methods. However, the application of HDR for particular allele or gene replacement in cotton remains challenging, and as of now, no stable lines with precise editing have been successfully obtained. To enhance HDR efficiency in cotton, several strategies used in other plant species and mammalian cells can be evaluated. One such strategy is the use of a modular RNA aptamer-streptavidin system to enrich the availability of donor repair templates (DRTs), which has shown significant improvements in HDR efficiency in human cells (Carlson-Stevermer et al., 2017).

Another approach involves increasing the temporal and spatial colocalization of DRTs and the Cas9 protein, which can be achieved through the use of SNAP-tag technology. Covalently tethering single-stranded donor oligonucleotides (ssODNs) to the Cas9/guide RNA ribonucleoprotein complex has also been shown to increase HDR efficiency by bringing the DRTs closer to the DNA double-strand break (DSB) site (Savic et al., 2018). Furthermore, utilizing tissue-specific promoters, such as the egg cell- and early embryo-specific DD45 gene promoter, can increase the efficiency of HDR-mediated genome editing. This is particularly relevant in cotton, as HDR take place with more frequency in egg cells (Miki et al., 2018; Wolter et al., 2018). Additionally, the development of a tandem repeat-HDR (TR-HDR) strategy (Lu et al., 2020), which involves the random insertion of a modified DNA fragment, shows promise for seamless in-locus tagging and fragment replacement in other crops and could potentially be applied to cotton. While these techniques show potential, further research is needed to optimize and adapt them specifically for cotton. Continued efforts to improve HDR efficiency in cotton will pave the way for precise gene/allele replacement and the development of novel germplasm with resistance to CLCuD.

### 8.2 Multiplex genome editing in cotton

Many agriculturally important traits, including yield per plant, are governed by a complex 827 genetic network or multiple genes. In order to understand the functions of these genes more thoroughly, expedite gene discovery, and facilitate breeding efforts, a proficient multiplex genome editing platform is essential. Multiplex genome editing enables the knockout of individual genes, combined genes, or specific gene regions, providing the means to dissect traits and support marker-assisted breeding. In cotton, the formulation of a proficient multiplex editing strategy is still in its early stages. However, insights from other plant species, such as wheat (Kumar et al., 2019) and *Arabidopsis* (Luo et al., 2021), can guide the optimization and systemic comparison of different vector components in cotton. For example, the use of robust constitutive promoters for the expression of Cas9 protein or guide RNA (gRNA), along with additional nuclear localization signals (NLSs), can contribute to the development of a more effective multiplex editing strategy.

Recent advancements in multiplex genome editing in wheat using a polycistronic tRNA strategy offer potential insights for cotton research. In an elite wheat variety, the simultaneous editing of multiple genes at up to 15 genomic loci was achieved by producing multiple mature single chimeric guide RNAs (sgRNAs) simultaneously. This approach utilized an expression cassette controlled by a rice Actin promoter and terminated by a Poly A sequence to enhance the transcripts stability (Luo et al., 2021). By adapting similar strategies to cotton, it may be possible to achieve efficient multiplex editing and accelerate gene discovery and breeding efforts. Expanding the horizons of multiplex editing in cotton can also involve the use of multiple Cas nucleases with different PAM requirements, like Cas12a, SaCas9, SpRY, SpG, xCas9 and SpCas9-NG (Zhang et al., 2019a; Jordan et al., 2021; Li et al., 2021). Additionally, multiplexing systems can be employed to target non-coding RNA regions and several other genetic regulatory elements, enabling the evaluation of their roles and further promoting cotton improvement against CLCuD. The development of a flexible and efficient CRISPR/Cas multiplexing system in cotton will highly facilitate fundamental biological research and translational breeding processes.

### 8.3 *De novo* domestication of wild species and site-directed artificial evolution of agriculturally important genes

Conventional breeding practices usually result in low stress resistance and loss of genetic diversity, posing challenges to crop stability and food security. To address these issues, the *de novo* domestication of wild species and site-directed artificial evolution of agriculturally important genes have emerged as potential approaches (Hickey et al., 2019). Through the comprehensive application of synthetic biology, genome editing, genomics and other cutting-edge technologies, wild species or landraces can be rapidly domesticated without disturbing the important traits (Eshed and Lippman, 2019). For instance, the introduction of traits of interest into stress-resilient wild tomato genotypes *via* multiplex genome editing resulted in offspring that displayed domesticated phenotypes while retaining salt tolerance and parental disease resistance (Li et al., 2018; Zsögön et al., 2018).

A rational approach for developing new crops varieties involves the *de novo* domestication of wild allotetraploid rice (*Oryza alta*, CCDD) has also been proposed. By editing several traits in *O. alta* and successfully producing allotetraploid rice lines with targeted improvements in agronomically important genes, researchers have demonstrated the potential for *de novo* domestication in cotton relatives or landraces (Yu et al., 2021). Additionally, base editing techniques could permit the artificial evolution of agriculturally significant genes in existing crop varieties, leading to the development of new germplasm with high genetic diversity. While these approaches have not been documented in cotton to date, the lessons learned from other crop species can inform future research and applications. The exploration of wild cotton species and related plant genomes, along with the manipulation of diverse gene modules, will provide cotton breeders with noval genes and modules for crop improvement. Comparative genomic analysis and in-depth studies of *Gossypium* species will contribute to the development of novel germplasm and the improvement of cotton varieties against CLCuD.

### 8.4 Development of genotype-independent and in planta delivery strategies

CRISPR-based genome editing techniques rely on the delivery of foreign DNA into plant cells to enable the function and expression of the CRISPR/Cas reagents. In cotton, this has traditionally been achieved through *Agrobacterium*-mediated transformation or biolistic transformation (Ribeiro et al., 2003; Rajasekaran, 2012). However, these methods are usually limited by low plant regeneration efficiency and the limited number of transformable genotypes, particularly in modern cotton varieties. To overcome these limitations, the development of genotype-independent and *in planta* delivery strategies is essential. A genotype-independent approach has been implemented in maize by introducing the Bbm expression cassette into embryos under the control of the maize auxin-inducible promoter (Lowe et al., 2018). This approach allows for the direct germination of embryos into plants without a callus phase, enabling more uniform and efficient transformation. Synthetic biology approaches, which involve the design and construction of new biological parts and systems, also offer potential solutions for improving cotton transformation. By utilizing synthetic biology principles, it may be possible to redesign the cotton genome to enhance transformation efficiency and broaden the range of transformable genotypes.

## 9 Concluding remarks

The future of controlling and developing resistance in cotton against CLCuD lies in the 893 application of advanced biotechnological and molecular breeding techniques. Cotton employs a range of defense mechanisms against CLCuD, including resistance genes, RNA interference, and natural defense chemicals. While controlling whitefly populations through insecticides is the primary method, biotechnological and molecular breeding approaches offer the most promising solutions for inducing resistance. NGS technologies have transformed the field of cotton genomics, enabling researchers to unravel the complexities of the cotton genome and understand the genetic basis of important traits. These technologies have opened up new avenues for studying gene expression, genetic variation, epigenomics, metagenomics, and functional genomics in cotton. With advancements in sequencing technologies and bioinformatics tools, the future of cotton genomics looks promising, paving the way for the development of improved cotton varieties with enhanced productivity, fiber quality, and disease resistance. Moreover, DNA methylation also plays a crucial role in cotton evolution, domestication, and disease resistance. Understanding the epigenetic regulation of CLCuD and the role of DNA methylation in cotton can provide valuable insights into the development of disease-resistant cotton varieties. The qPCR analysis of virus and satellite DNA levels can be a useful tool for assessing cotton yield and selecting resistant varieties for cultivation. Future research should focus on unraveling the complex interactions between DNA methylation, gene expression, disease resistance and genome editing in cotton to develop sustainable strategies fo disease management in cotton cultivation. Precision genome editing *via* HDR holds promise for achieving specific allele/gene replacement and developing resistance against CLCuD. Similarly, multiplex genome editing offers opportunities for MAS, gene discovery and trait dissection. The newly domesticated wild species and site-directed artificial evolution of agriculturally important genes provide avenues for enhancing genetic diversity and developing novel germplasm. Additionally, the formulation of genotype-independent and *in planta* delivery strategies, along with the utilization of synthetic biology principles, can overcome the limitations of traditional transformation methods. Furthermore, speed breeding has also revolutionized the field of plant breeding, offering breeders a powerful tool to speed-up the development of new crop varieties. In the case of cotton, speed breeding holds immense promise for combating Cotton Leaf Curl Disease and addressing other agronomic challenges. By leveraging the advantages of specialized lighting solutions and controlled growth environments, breeders can expedite the breeding process, enhance disease resistance, improve fiber quality, increase yield, and develop climate-resilient cotton varieties. With companies like Nexsel leading the way in providing cutting-edge speed breeding solutions, the future of cotton breeding looks brighter than ever.

As these techniques continue to be optimized and adapted specifically for cotton, they have the potential to revolutionize cotton improvement and pave the way for sustainable cotton production. By developing cotton varieties with enhanced resistance against CLCuD, the devastating impacts of this disease can be mitigated, leading to increased yields and improved livelihoods for cotton farmers. The future of cotton research is bright, with biotechnological advancements driving innovation and unlocking the full potential of this important fiber crop.
